# Classification of positive blood cultures: computer algorithms versus physicians' assessment - development of tools for surveillance of bloodstream infection prognosis using population-based laboratory databases

**DOI:** 10.1186/1471-2288-12-139

**Published:** 2012-09-12

**Authors:** Kim O Gradel, Jenny Dahl Knudsen, Magnus Arpi, Christian Østergaard, Henrik C Schønheyder, Mette Søgaard

**Affiliations:** 1 Research Unit of Clinical Epidemiology, Institute of Clinical Research, University of Southern Denmark, Odense, Denmark; 2 Centre for National Clinical Databases, South, Odense University Hospital, Odense, Denmark; 3 Department of Clinical Microbiology, Copenhagen University Hospital Hvidovre Hospital, Hvidovre, Denmark; 4 Department of Clinical Microbiology, Copenhagen University Hospital Herlev Hospital, Herlev, Denmark; 5 Department of Clinical Microbiology, Aalborg Hospital, Aarhus University Hospital, Aalborg, Denmark

## Abstract

**Background:**

Information from blood cultures is utilized for infection control, public health surveillance, and clinical outcome research. This information can be enriched by physicians’ assessments of positive blood cultures, which are, however, often available from selected patient groups or pathogens only. The aim of this work was to determine whether patients with positive blood cultures can be classified effectively for outcome research in epidemiological studies by the use of administrative data and computer algorithms, taking physicians’ assessments as reference.

**Methods:**

Physicians’ assessments of positive blood cultures were routinely recorded at two Danish hospitals from 2006 through 2008. The physicians’ assessments classified positive blood cultures as: a) contamination or bloodstream infection; b) bloodstream infection as mono- or polymicrobial; c) bloodstream infection as community- or hospital-onset; d) community-onset bloodstream infection as healthcare-associated or not. We applied the computer algorithms to data from laboratory databases and the Danish National Patient Registry to classify the same groups and compared these with the physicians’ assessments as reference episodes. For each classification, we tabulated episodes derived by the physicians’ assessment and the computer algorithm and compared 30-day mortality between concordant and discrepant groups with adjustment for age, gender, and comorbidity.

**Results:**

Physicians derived 9,482 reference episodes from 21,705 positive blood cultures. The agreement between computer algorithms and physicians’ assessments was high for contamination vs. bloodstream infection (8,966/9,482 reference episodes [96.6%], Kappa = 0.83) and mono- vs. polymicrobial bloodstream infection (6,932/7,288 reference episodes [95.2%], Kappa = 0.76), but lower for community- vs. hospital-onset bloodstream infection (6,056/7,288 reference episodes [83.1%], Kappa = 0.57) and healthcare-association (3,032/4,740 reference episodes [64.0%], Kappa = 0.15). The 30-day mortality in the discrepant groups differed from the concordant groups as regards community- vs. hospital-onset, whereas there were no material differences within the other comparison groups.

**Conclusions:**

Using data from health administrative registries, we found high agreement between the computer algorithms and the physicians’ assessments as regards contamination vs. bloodstream infection and monomicrobial vs. polymicrobial bloodstream infection, whereas there was only moderate agreement between the computer algorithms and the physicians’ assessments concerning the place of onset. These results provide new information on the utility of computer algorithms derived from health administrative registries.

## Background

Bloodstream infection is a serious infection defined by the presence of viable bacteria or fungi in the bloodstream as evidenced by positive blood cultures. In patients with positive blood cultures it has clinical priority to assess whether a positive blood culture represents contamination or bloodstream infection [1,2]. Further classifications address whether the infection is monomicrobial or polymicrobial [1,3,4] and whether the site of acquisition is inside or outside the hospital setting [5,6]. These classifications are important because they are closely related to risk factors for bloodstream infection, the site of the infection in the body, the nature of the microbial agent, antibiotic resistance, and prognosis [1,7].

Classification of positive blood cultures is traditionally based on all available clinical and microbiological information and it is performed by physicians using standardized definitions [5,6,8]. This is an integral part of clinical decision making but the classifications are rarely recorded in a systematic way. Hence, it is labor intensive to retrieve these data and they are often available only for selected patient groups (e.g., in the intensive care unit or for specific pathogens). This constitutes a barrier to both surveillance and research. However, computer algorithms that utilize existing laboratory and clinical data from health administrative registries may facilitate infection surveillance and clinical outcome research [9]. Currently, the increasing levels of antibiotic resistance underscore the need for effective and timely monitoring of outcomes in patients with community- or hospital-onset bloodstream infection [10].

Previous studies have used computer algorithms, primarily based on laboratory data, to define the above classifications [8,11-17]. Few of these, however, compared their computer algorithms with the physicians’ assessments, and they only comprised contamination vs. bloodstream infection [8,13-15] or community- vs. hospital-onset [8,12,17]. None of the studies evaluated whether possible misclassifications were non-differential or differential, for instance by assessing their utility for the monitoring of prognosis.

In Denmark, physicians in departments of clinical microbiology assess each patient’s positive blood cultures and notify attending physicians. In two large hospitals, the physicians’ assessments of all positive blood cultures have been recorded electronically during a 3-year period. These data enabled this study in which we derived computer algorithms to classify positive blood cultures and compared the performance of these with the results of the physicians’ assessments. To examine whether possible misclassifications were differential, we further compared 30-day mortality (a commonly used outcome in prognostic studies) for patients with blood cultures classified by physicians and the algorithms. The overall aim was to determine whether the combined use of health administrative data and computer algorithms would be an effective tool for outcome research in epidemiological studies.

## Methods

### Setting

Herlev Hospital and Hvidovre Hospital are situated in the Capital Region of Denmark. Microbiological diagnostic service is provided by each hospital’s Department of Clinical Microbiology, at Herlev to all clinical wards in Herlev Hospital and two other hospitals (Gentofte and Glostrup) and at Hvidovre to all clinical wards in Hvidovre Hospital and four other hospitals (Bispebjerg, Frederiksberg, Amager, and Bornholm). The terms Herlev and Hvidovre are used onwards to denote each department and the hospitals it serves.

The Danish health-care system is financed through the tax system and provides care free of charge for all residents. Acutely ill patients are admitted to the nearest hospital in their region of residence. During the study period, Herlev and Hvidovre had an average background population of 620,000 and 635,000, respectively [18].

### General principles for data linkage

All Danish residents have a unique personal identification number (the Civil Registration Number, which incorporates date of birth and gender) used for all health contacts, that permits linkage between health administrative registries [19].

### Blood culture procedures

The ordering of blood cultures was based on the attending physician’s clinical assessment. Blood cultures are rarely ordered by general practitioners and were not considered in this study. The target of blood sample volume was 30–40 mL (2 x 2 bottles comprising a blood culture set) from adults and teenagers, and 0.5-3 mL from children (1 bottle comprising a blood culture set). The BACTEC 9240^TM^ blood culture system (Becton Dickinson, Sparks, MD, USA) was used at Herlev and the BacT/Alert^TM^ blood culture system (bioMérieux, Marcy l’Etoile, France) at Hvidovre. Bornholm Hospital, however, performed its own blood culture procedures, using the BACTEC 9240^TM^ blood culture system. Positive blood cultures were immediately examined by Gram stain and wet mount microscopy and subcultured onto plate media selected in accordance with the Gram stain result. Isolates were routinely identified by a combination of conventional and commercial methods.

### Blood culture data

Both Herlev and Hvidovre used the electronic laboratory information system ADBakt (Autonik, Sköldinge, Sweden) for the recording of the blood culture results. Blood culture isolates were normally recorded by the species name; in instances of obvious contamination or when only one amongst several isolates was speciated a provisional name or grouping was used (e.g., coryneform rod, coagulase-negative staphylococcus, or yeast-like organism). Our preliminary study database included all positive blood cultures at the two departments of clinical microbiology from 2005 through 2008. Herlev numbered a positive blood culture (one observation in the database, Table 1) per bacterial species per blood culture bottle, whereas Hvidovre numbered a positive blood culture per bacterial species per 2 bottles within the same blood culture set. Thus, for adults a monomicrobial blood culture set from Herlev could have up to 4 positive blood cultures and a monomicrobial blood culture set from Hvidovre could have up to 2 positive blood cultures. For polymicrobial blood culture sets an indefinite number of positive blood cultures was theoretically possible. Because the number system did not enable us to determine which blood culture bottles belonged to the same blood culture set we used date as the “preliminary analytical unit”. The important variables for our study were the dates of draw and receipt of the blood culture and the isolated microorganism(s). The date of draw was available for 21,907 of the 24,028 positive blood cultures (91.2%), whereas the date of receipt was available for all blood cultures. Therefore, we compiled a best-estimate-date, defined as the date of draw, and if this date was missing, the date of receipt (Table 1).

**Table 1:** Definitions of key terms in the computer algorithms. Variables are in italics

**Term**	**Definition**
Positive blood culture	One observation (row) in the database
*Best-estimate-date*	Date of draw of blood culture. If date of draw of blood culture is missing: date of receipt of blood culture (never missing)
The patient’s first computer episode	All positive blood cultures on the patient’s earliest *best-estimate-date* and the day after the earliest *best-estimate-date*
The patient’s subsequent computer episodes after the first computer episode	First available date after the first computer episode and the day after (second computer episode), first available date after the second computer episode and the day after (third computer episode), etc.
Contamination computer episode	Only common skin commensals (coagulase-negative staphylococci, *Propionibacterium* spp., *Bacillus* spp., *Micrococcus* spp., or *Corynebacterium* spp.) were detected on only the earliest *best-estimate-date* of the computer episode within a 5-day period and no pathogens were detected in the computer episode
Bloodstream infection computer episode	A computer episode that is not a contamination computer episode
Monomicrobial bloodstream infection computer episode	Only 1 type of microorganism isolated within the bloodstream infection computer episode
Polymicrobial bloodstream infection computer episode	≥ 2 types of microorganism isolated within the bloodstream infection computer episode
Inpatient contact	A contact recorded in the Danish National Patient Registry (cf. text) in which the patient is hospitalized
Outpatient contact	An ambulatory or emergency room contact in the Danish National Patient Registry (cf. text)
*indate*	Earliest date of contact, as recorded in the Danish National Patient Registry (cf. text). For an inpatient contact, the date of admission, either from the home or from another hospital ward
*outdate*	Latest date of contact, as recorded in the Danish National Patient Registry (cf. text). For an inpatient contact, the date of discharge, either to the home or to another hospital ward
*time_in*	*Best-estimate-date* minus *indate* (computed for all combinations of *best-estimate-date* and *indate* for each patient, *time_in* < −2 days omitted)
*time_out*	*Best-estimate-date* minus *outdate* (computed for all combinations of *best-estimate-date* and *outdate* for each patient, *time_out* > 30 days omitted)
Hospital-onset bloodstream infection computer episode	For patients admitted from home to the ward in which the blood culture was retrieved: Bloodstream infection computer episode where the lowest *time_in* among inpatient contacts is ≥ 2 days
	Or, for patients admitted from another ward to the ward in which the blood culture was retrieved:
	(Bloodstream infection computer episode where the lowest *time_in* among inpatient contacts is 0 days or 1 day) and (≥ 1 inpatient contacts within the computer episode has *time_out* = 0 days combined with either *time_out* minus *time_in* ≥ 2 days [if time_in = 0 days] or with *time_out* minus *time_in* ≥ 1 day [if time_in = 1 day])
	or
	(Bloodstream infection computer episode where the lowest *time_in* among inpatient contacts is 0 days or 1 day) and (≥ 1 inpatient contact within the computer episode has *time_in* > 1 day and *time_out* < 0 days)
Community-onset bloodstream infection computer episode	Bloodstream infection computer episode where its lowest *time_in* among inpatient contacts is 0 days or 1 day and the computer episode is not hospital-onset
Healthcare-associated computer episode	A community-onset computer episode with (*time_in* ≤ 30 days and *time_out* > 0 days) or (*time_in* > 30 days and 30 days ≥ *time_out* > 0 days). *time_in* and *time_out* are computed for both inpatient and outpatient contacts

### Linkage to other data sources

The Danish National Patient Registry includes all somatic inpatient contacts since 1977 and all somatic outpatient contacts (ambulatory and emergency room visits) since 1995. For each contact, it includes date of admission and discharge and up to 20 discharge diagnoses coded according to the International Classification of Diseases (ICD) system, ICD-8 in 1977–1993 and ICD-10 thereafter [20]. Data from the Danish National Patient Registry were used to derive computer algorithms and to identify the first-time occurrence (since 1977 up to the best-estimate-date) of selected comorbid diseases included in the Charlson comorbidity index [21]. In this prognostic index, 19 major disease categories (e.g., malignancy, cardiovascular diseases, and diabetes mellitus) are assigned a score, with higher scores given to more severe diseases.

To enable follow-up, we linked our data to the Danish Civil Registration System, which contains daily updated records on the vital status of all Danish residents, including date of death or emigration [22].

### Derivation of episodes from the physicians’ assessments

Since 2006, physicians in the two departments of clinical microbiology have recorded clinical data in a predefined electronic form concurrently with the oral notification of each blood culture thought to define a contamination or a new bloodstream infection episode. These physicians’ assessments, for which there were no formally specified criteria, were made in cooperation with the attending physicians in the patient’s clinical ward. We linked these data to the study database.

We used two variables in the recorded physicians’ assessments. The first variable determined whether the positive blood culture was part of a contamination or a bloodstream infection episode and for the bloodstream infections whether they had a community-onset, were healthcare-associated, or had a hospital-onset. The second variable also distinguished between a contamination and a bloodstream infection episode, but further determined whether the bloodstream infection was monomicrobial or polymicrobial.

For each patient in the period 2006–2008, the positive blood culture with the earliest best-estimate-date and a recorded physicians’ assessment determined the patient’s first reference episode. We then determined the patient’s subsequent reference episode to be the next positive blood culture on a subsequent date with a physicians’ assessment recorded. This was reiterated until all possible physicians’ assessment-derived episodes were computed for all patients. All positive blood cultures with no recorded physicians’ assessment, which occurred within 30 days after the earliest best-estimate-date with a recorded physicians’ assessment, were included in the reference episode.

### Derivation of computer algorithms

Definitions of the key terms in the computer algorithms are given in Table 1. The statistical codes for the computer algorithms, written in Stata® do-files, may be obtained from the corresponding author.

The patient’s first computer episode comprised all positive blood cultures on the patient’s earliest best-estimate-date and the day after. The next computer episode was computed from the next available best-estimate-date and the day after and so forth.

We defined a contamination computer episode using the criteria defined by Trick et al. [8], as detailed in Table 1.

We defined a bloodstream infection computer episode as polymicrobial if more than one type of microorganism was isolated within the computer episode.

To classify the place of onset, we combined all best-estimate-dates for each bloodstream infection computer episode with all the patient’s inpatient contacts recorded in the Danish National Patient Registry since 2005. We omitted all inpatient contact records which occurred more than 30 days before or two days after the best-estimate-date. For each bloodstream infection computer episode we computed the shortest time period between admission from home and the best-estimate-date. If this time period was 0 or 1 day the bloodstream infection computer episode was classified as community-onset and if it was ≥2 days it was classified as hospital-onset [6].

To determine whether bloodstream infections with community-onset were healthcare-associated, we recombined all the community-onset computer episodes to the Danish National Patient Registry as described above, but further included outpatient contacts (i.e., ambulatory and emergency room contacts). A healthcare-associated computer episode was defined as a community-onset computer episode with an inpatient or outpatient contact in the 30-day period up to the earliest best-estimate-date [7].

### Incident and non-incident reference episodes

We used 2005 as a lag year to decide whether the first reference episode during 2006–2008 was ‘truly’ incident. That is, if the patient had one or more positive blood cultures recorded in 2005 within 365 days prior to the first-time positive blood culture in 2006, we characterized the first-time reference episode in 2006–2008 as ‘not truly’ incident.

### Statistical analyses

The reference episode, with its positive blood cultures, was the analytical unit.

Initially, we compared age, gender, Charlson comorbidity score (0, 1–2, and >2), and 30-day mortality (yes vs. no) between reference episodes and computer episodes without reference episodes, including all patients and excluding patients who had both reference episodes and computer episodes without reference episodes. We used the Wilcoxon rank-sum test to compare age and the Chi-square test to compare the categorical variables.

We computed 2x2 contingency tables comparing the following physicians’ assessments and computer algorithms: a) contamination vs. bloodstream infection; b) monomicrobial vs. polymicrobial bloodstream infection; c) community-onset vs. hospital-onset bloodstream infection; d) community-onset bloodstream infection: healthcare-association vs. no healthcare-association. For each comparison we evaluated the accuracy by computing agreement percentages between the physicians’ assessment- and the computer algorithm-derived groups as well as the Kappa-value [23]. Positive Kappa-values were categorized into 0–0.2 (slight agreement), 0.2-0.4 (fair agreement), 0.4-0.6 (moderate agreement), 0.6-0.8 (substantial agreement), and 0.8-1.0 (almost perfect agreement) [23].

For the four comparisons listed above we computed Kaplan-Meier mortality curves up to 30 days after the best-estimate-date. For each comparison we used logistic regression analyses with odds ratios (ORs) and 95% confidence intervals (CIs) to assess 30-day mortality for the concordant and discrepant groups. Within each comparison we used the concordant group presumed to be prognostically worst (bloodstream infection, polymicrobial, hospital-onset, and healthcare-association, respectively) as reference and conducted crude analyses and analyses adjusted for age (continuous variable), gender, and comorbidity (Charlson comorbidity score 0, 1–2, and >2).

Finally, we reiterated all analyses in subgroups (Herlev, Hvidovre, incident, and non-incident reference episodes) to estimate whether results in these subgroups differed from the overall results.

The program Stata®, vs. 11 (StataCorp, College Station, TX, USA) was used for all analyses.

### Ethical considerations

The study was conducted according to the guidelines of the regional scientific ethics committee for use of clinical and laboratory data and approved by the Danish Data Protection Agency (record no. 2007-41-0627).

## Results

### Descriptive data

**Figure 1:**
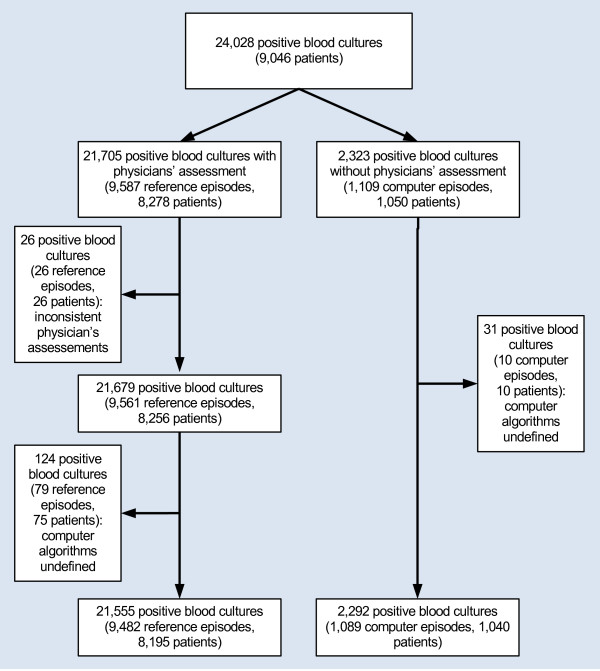
**Flowchart of positive blood cultures, reference episodes, computer episodes, and patients.** For definitions, see Table 1. As the same patients may appear in different categories, the number of patients does not necessarily correlate with differences between boxes.

A total of 24,028 positive blood cultures were recorded from 2006 through 2008 and from 21,555 (89.7%) of these we derived 9,482 reference episodes (Figure 1). From 2,292 of the remaining 2,323 blood cultures (98.7%) 1,089 computer episodes were computed. It was mainly at the beginning of the registration period that fewer blood cultures were assessed by physicians (487/1,089 computer episodes (44.7%) from January through April 2006, data not shown). We compared reference episodes and computer episodes without reference episodes pertaining to age, gender, comorbidity, and 30-day mortality (Table 2). There were fewer females amongst computer episodes without reference episodes, but the exclusion of the 280 patients who had both reference episodes and computer episodes without reference episodes rendered a more equal gender distribution. Notwithstanding this exclusion, patients having computer episodes without reference episodes had higher 30-day mortality. The 8,195 patients with reference episodes experienced from 1 to 10 reference episodes (Table 3)

**Table 2:** Characteristics of reference episodes and computer episodes without reference episodes

**Text**	**Reference episodes**	**Computer episodes without reference episodes**	**p-value**
All episodes, number	9,482	1,089	
Age, years	71.6 (58.1-81.7)^1^	70.2 (58.5-81.7)	0.54
Females	4,531 (47.8)^2^	483 (44.4)	0.03
Charlson comorbidity score^3^			0.33
0	2,370 (25.0)	252 (23.1)	
1-2	3,449 (36.4)	396 (36.4)	
>2	3,663 (38.6)	441 (40.5)	
30-day mortality	1,998 (21.1)^4^	284 (26.1)^5^	0.0001
Separate episodes^6^, number	9,102	796	
Age, years	71.6 (58.1-81.7)	69.6 (58.2-81.2)	0.16
Females	4,382 (48.1)	368 (46.2)	0.30
Charlson comorbidity score			0.85
0	2,309 (25.4)	197 (24.8)	
1-2	3,319 (36.5)	298 (37.4)	
>2	3,474 (38.2)	301 (37.8)	
30-day mortality	1,926 (21.2)^4^	233 (29.3)^5^	0.0001

^1^ Median (25^th^-75^th^ percentile); ^2^ Number (percent); ^3^ Cf. [21] and text; ^4^ Could not be assessed for 10 reference episodes; ^5^ Could not be assessed for 1 episode; ^6^ Exclusion of the 280 patients who had reference episodes and computer episodes without reference episodes.

**Table 3:** Number of reference episodes per patient

**Number of reference episodes**	**Number of patients (%)**
1	7,223 (88.1)
2	762 (9.3)
3	141 (1.7)
4	44 (0.5)
5	14 (0.2)
6	6 (0.1)
7	1(0)
8	3 (0)
9	0 (0)
10	1 (0)
Total	8,195 (100)

### Bloodstream infection vs. contamination

Of the 9,482 reference episodes, 7,288 (76.9%) were classified as bloodstream infection and 1,678 (17.7%) as contaminations by both the physicians’ assessment and the computer algorithm (Table 4). The Kappa-value of 0.83 indicated almost perfect agreement between the physicians’ assessment- and the computer algorithm-derived groups.

**Table 4:** Distribution of reference episodes according to the physicians’ assessments and the computer algorithms

		Physicians’ assessment			
**All reference episodes**		Bloodstream infection	Contamination	Total	Kappa
Computer algorithm	Bloodstream infection	7,288 (76.9)^1^	276 (2.9)	7,564	0.83
	Contamination	240 (2.5)	1,678 (17.7)	1,918	
	Total	7,528	1,954	9,482	
**Bloodstream infection reference episodes from both assessments**		Polymicrobial	Monomicrobial		
Computer algorithm	Polymicrobial	638 (8.8)	305 (4.2)	943	0.76
	Monomicrobial	51(0.7)	6,294 (86.4)	6,345	
	Total	689	6,599	7,288	
**Bloodstream infection reference episodes from both assessments**		Community	Hospital		
Computer algorithm	Community	4,740 (65.0)	289 (4.0)	5,029	0.57
	Hospital	943 (12.9)	1,316 (18.1)	2,259	
	Total	5,683	1,605	7,288	
**Community-onset bloodstream infection reference episodes from both assessments**		Healthcare-associated	Not healthcare-associated		
Computer algorithm	Healthcare-associated	357 (7.5)	1,547 (32.6)	1,904	0.15
	Not healthcare-associated	161 (3.4)	2,675 (56.4)	2,836	
	Total	518	4,222	4,740	

^1^ Number of reference episodes (percentage of table total).

### Monomicrobial vs. polymicrobial bloodstream infection

The Kappa-value was 0.76, denoting substantial agreement (Table 4). Most of the reference episodes in the discrepant groups were classified by the computer algorithm as polymicrobial, but by the physicians’ assessment as monomicrobial.

### Community-onset vs. hospital-onset bloodstream infection

There was a moderate agreement (Kappa = 0.57) between the physicians’ assessment and the computer algorithm (Table 4). Among the 16.9% of the reference episodes in the discrepant groups, 943 (76.5%) were classified as hospital-onset by the computer algorithm and community-onset by the physicians’ assessment.

### Healthcare-associated community-onset bloodstream infection

This comparison rendered the lowest Kappa-value of 0.15, which indicates only slight agreement (Table 4). In the discrepant groups, 1,547 of 1,708 reference episodes (90.6%) were classified as healthcare-associated according to the computer algorithm, but not the physicians’ assessment.

### 30-day mortality

30-day follow-up was possible for all but 10 of the 9,482 reference episodes. The differences between the crude and the adjusted models were generally minor (Table 5).

**Table 5:** 30-day mortality analyses, using logistic regression analyses

**Comparison**	**Physicians’ assessment**	**Computer algorithm**	**Odds ratios (95% confidence intervals)**	
			**Crude model**	**Adjusted model**^**1**^
Bloodstream infection vs. contamination	Bloodstream infection	Bloodstream infection	1 (reference)	1 (reference)
	Contamination	Bloodstream infection	0.94 (0.70-1.27)	1.18 (0.87-1.60)
	Bloodstream infection	Contamination	0.89 (0.65-1.23)	1.04 (0.75-1.45)
	Contamination	Contamination	0.74 (0.64-0.85)	0.84 (0.73-0.97)
Polymicrobial vs. monomicrobial	Polymicrobial	Polymicrobial	1 (reference)	1 (reference)
	Monomicrobial	Polymicrobial	0.76 (0.56-1.03)	0.78 (0.57-1.07)
	Polymicrobial	Monomicrobial	1.15 (0.63-2.09)	1.19 (0.64-2.22)
	Monomicrobial	Monomicrobial	0.55 (0.46-0.66)	0.56 (0.47-0.67)
Hospital-onset vs. community-onset	Hospital-onset	Hospital-onset	1 (reference)	1 (reference)
	Community-onset	Hospital-onset	0.66 (0.55-0.79)	0.65 (0.54-0.78)
	Hospital-onset	Community-onset	0.60 (0.45-0.80)	0.63 (0.47-0.86)
	Community-onset	Community-onset	0.40 (0.35-0.46)	0.40 (0.35-0.46)
Healthcare-association vs. no healthcare-association	Healthcare-association	Healthcare-association	1 (reference)	1 (reference)
	No healthcare-association	Healthcare-association	1.26 (0.94-1.68)	1.20 (0.89-1.62)
	Healthcare-association	No healthcare-association	0.80 (0.48-1.32)	0.77 (0.46-1.29)
	No healthcare-association	No healthcare-association	0.74 (0.56-0.99)	0.77 (0.57-1.04)

^1^ Adjusted for age, gender, and comorbidity.

**Figure 2:**
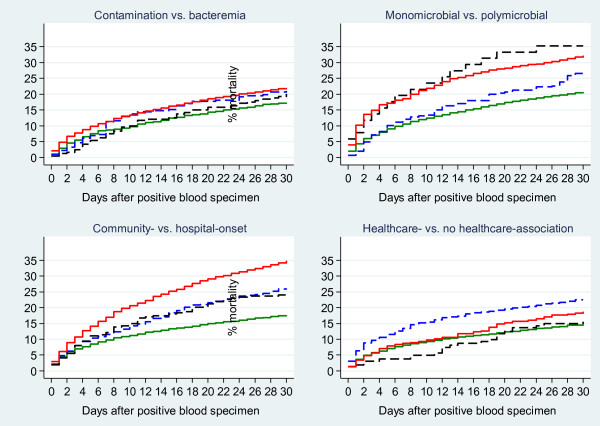
**Kaplan-Meier mortality curves up to day 30 for patient groups derived from computer algorithms or physicians’ assessments.** Contamination vs. bloodstream infection: contamination according to computer algorithm and physicians’ assessment (green solid line); bloodstream infection according to computer algorithm and physicians’ assessment (red solid line); contamination according to computer algorithm, bloodstream infection according to physicians’ assessment (black dashed line); bloodstream infection according to computer algorithm, contamination according to physicians’ assessment (blue dashed line). Monomicrobial vs. polymicrobial: monomicrobial according to computer algorithm and physicians’ assessment (green solid line); polymicrobial according to computer algorithm and physicians’ assessment (red solid line); monomicrobial according to computer algorithm, polymicrobial according to physicians’ assessment (black dashed line); polymicrobial according to computer algorithm, monomicrobial according to physicians’ assessment (blue dashed line). Community- vs. hospital-onset: community-onset according to computer algorithm and physicians’ assessment (green solid line); hospital-onset according to computer algorithm and physicians’ assessment (red solid line); community-onset according to computer algorithm, hospital-onset according to physicians’ assessment (black dashed line); hospital-onset according to computer algorithm, community-onset according to physicians’ assessment (blue dashed line). Healthcare- vs. no healthcare-association: no healthcare-association according to computer algorithm and physicians’ assessment (green solid line); healthcare-association according to computer algorithm and physicians’ assessment (red solid line); no healthcare-association according to computer algorithm, healthcare-association according to physicians’ assessment (black dashed line); healthcare-association according to computer algorithm, no healthcare-association according to physicians’ assessment (blue dashed line).

For classification of contamination vs. bloodstream infection, reference episodes classified as contamination by both the computer algorithm and physicians’ assessment had the lowest relative 30-day mortality compared with the bloodstream infection concordant group, whereas mortality for the two discrepant groups was similar to the bloodstream infection concordant group (Figure 2, Table 5).

Mortality in patients with bloodstream infection classified as monomicrobial by both the computer algorithm and the physicians’ assessment was lower compared with patients having bloodstream infection classified as polymicrobial by both. None of the discrepant groups differed materially from the polymicrobial concordant group, though CIs for the physicians’ assessment polymicrobial/computer algorithm monomicrobial group were wide due to few reference episodes (Figure 2, Table 5).

Both patients with concordant community-onset bloodstream infection and the two discrepant groups had lower mortality than patients with concordant hospital-onset bloodstream infection (Figure 2, Table 5). Still, both discrepant groups had higher mortality than the concordant community-onset bloodstream infection group.

Patients in the concordant group with no healthcare-association tended to have lower mortality than patients in the concordant healthcare-association group, whereas the two discrepant groups did not differ from the latter (Figure 2, Table 5).

### Subgroup analyses

There were 5,334 reference episodes at Herlev and 4,148 at Hvidovre. A total of 8,189 (86.4%) of the 9,482 reference episodes were incident. As only 79 (0.96%) of these had one or more positive blood cultures within 365 days prior to the first-time positive blood culture in 2006 (data not shown), there were no material differences between the number of ‘truly’ and ‘not truly’ incident reference episodes.

Analyses in all subgroups (Herlev, Hvidovre, incident, and non-incident reference episodes) did not differ notably from the overall results (data not shown).

## Discussion

We compared the classification of positive blood cultures by different computer algorithms with the assessments made by physicians in two departments of clinical microbiology. The highest agreement between the computer algorithms and the physicians’ assessments was seen for contamination vs. bloodstream infection and monomicrobial vs. polymicrobial bloodstream infection. In contrast, agreement in relation to the onset of the bloodstream infection performed less well, especially in relation to healthcare-association.

Short-term mortality is an important outcome in prognostic bloodstream infection studies, in which the place of onset is often included as a possible predictor [9]. We used the 30-day mortality to evaluate whether patients in the discrepant groups differed from patients in the concordant groups assumed to have the worst prognosis (bloodstream infection, polymicrobial bloodstream infection, hospital-onset bloodstream infection, and healthcare-associated community-onset bloodstream infection). The main reason for applying this commonly used outcome was to evaluate whether possible misclassifications were differential (i.e., related to the outcome). If not, the impact of the misclassification will cause the real estimates to deviate towards the null-hypothesis, which is a less severe bias than a differential misclassification. The use of other analyses and parameters (e.g., the distribution of antibiotic resistant bacteria in the groups, as well as other covariates than age, gender, and comorbidity) could also elucidate such aspects, but for these principal results we opted for a prognostic model with short-term mortality as the outcome.

The distinction between community- and hospital-onset bloodstream infection should optimally be based on individual assessment of all available clinical information as originally pointed out by Garner et al. [5]. Specific time windows have proved to be effective tools especially for infection control purposes, but there is little evidence to support discriminative time windows [24].

Patients with community-onset bloodstream infection often have healthcare-association, which is a predictor of a worse prognosis [6]. In the literature, healthcare-association includes home therapy, residence in a nursing home, or hospital contact before the actual admission [6]. However, physicians at Herlev and Hvidovre only evaluated prior hospital contacts, and not residence in a nursing home or home therapy, in relation to whether a reference episode was healthcare-associated or not. Still, it was unanticipated that our computer algorithm, which only captured prior hospital contacts, generated 1,904 healthcare-associated reference episodes, whereas the physicians’ assessment generated just 518 reference episodes. It may lead to the question whether physicians have difficulties in discovering patients’ recent hospital contacts when doing their assessment. In comparison, the computer algorithm has the advantage of access to the Danish National Patient Registry which records all hospital admissions and ambulatory contacts.

The definition of monomicrobial vs. polymicrobial bloodstream infection is closely linked to the definition of a bloodstream infection episode per se. There is no general agreed-upon definition, although there has been wide acceptance of assigning two or more microorganisms of different species or type to one episode if blood cultures are taken within a 2-day time window as done in this study. Still, it may be debatable whether the isolation of additional microorganisms represents a super-infection or a new episode [3,25]. Moreover, the computer algorithm probably over-estimated a few polymicrobial reference episodes because of very rare recordings of microorganisms not identified on species level (reference episodes in which only one among several isolates was speciated). Thus, a reference episode with both *Candida albicans* and ‘yeast-like organisms’ will be classified as polymicrobial according to the computer algorithm. Nevertheless, we found a high level of agreement between the computer algorithm and the physicians’ assessment.

We are aware of only four previous studies comparing computer algorithms and clinical assessments for the distinction between contamination and bloodstream infection (all of which comprised only common skin commensals or coagulase-negative staphylococci) [8,13-15] and three studies distinguishing between community- and hospital-onset [8,12,17]. Comparison of the results to our findings is difficult for several reasons. All the studies were prospective and used clinical assessments specifically designed for the given study. Moreover, various computer algorithm criteria (including combinations with clinical data) were used and the settings, none of which were population-based, differed from ours.

Our study had several strengths. The high number of patients enabled a high statistical precision and stratified analyses, follow-up was possible for virtually all patients, it was population based, and included numerous hospital wards. Moreover, the physicians’ assessments reflected clinical assessments performed on a daily routine basis and was thus unrelated to the research question.

There were, however, also important limitations. First, the data were based on numerous physicians’ assessments performed as part of the clinical decision making, with no evaluation of inter-observer agreement or use of formally specified criteria. This is in contrast to other studies in which prospectively defined criteria were used specifically for the given study [8,12-15,17]. Second, because some of the patients were included more than once the observations were not interdependent, e.g., related to short-term mortality. Nevertheless, we selected all, and not only incident, episodes to evaluate the clinical assessments, which are principally performed for all positive blood cultures. The high number of episodes enabled subgroup analyses comparing incident and non-incident episodes, which did not reveal any material differences. Third, the clustering of computer episodes without reference episodes at the beginning of the registration period and the equal age and comorbidity distribution between patients with and without reference episodes indicated that patients with reference episodes constituted a non-selective group. However, the higher mortality amongst patients without reference episodes indicates that physicians tended to omit the assessment of these patients as this was clinically less relevant, which has also been reported from other Danish studies [26,27]. Finally, the laboratory data had some flaws: lack of date of draw for some blood cultures, recording of time of draw and receipt of the blood cultures only by date (and not hour and minute), no possibility to categorize blood culture sets from specimen identification numbers, and lack of speciation of a few microorganisms. Altogether, these limitations probably had only minor impact on the overall results.

Though we cannot characterize the physicians’ assessments as gold standard the high agreements and the correspondingly high Kappa values for bloodstream infection vs. contamination and monomicrobial vs. polymicrobial bloodstream infection indicate that our computer algorithms can be used to robustly categorize these groups. With regard to place of onset we believe that computer algorithms cannot replace the physicians’ assessments and further studies using various time windows and more standardized clinical assessments are needed before firmer conclusions can be reached.

Laboratory-confirmed bloodstream infection is one of the best defined groups of bacterial infections and is associated with high 30-day mortality, as appears from Figure 2. The European Antimicrobial Resistance Surveillance System (EARSS), which is now managed by the European Centre for Disease Prevention and Control, estimated in 2007 that 2.8 million blood cultures were obtained from a population of 120 million European citizens in that year [28]. For all of Europe this is equivalent to more than 10 million blood cultures. Effective classification of positive blood cultures, especially the distinction of hospital- and community-onset cases, can be of great value for both community health and hospital infection surveillance. However, information on the utility of computer algorithms for surveillance and research in patients with bloodstream infection is scarce. On this background, further refinement and validation of computer algorithms must be given high priority.

## Conclusions

We tested computer algorithms in comparison to prospectively and routinely recorded clinical assessment for a high number of patients with positive blood cultures in a population based setting. Our computer algorithms showed a high level of agreement with the physicians’ assessments for distinguishing between contamination and bloodstream infection and between monomicrobial and polymicrobial bloodstream infection. In contrast, agreement regarding place of onset was lower, which is not surprising given the somewhat arbitrary distinction between community- and hospital-onset [5,6]. Prognostic short term mortality models may thus differ according to whether they are based on computer algorithm- or physicians’ assessment-derived data on the place of onset. These preliminary results have implications for studies using data from health administrative databases; future studies that retrieve data from medical records and refine computer algorithms are needed.

## Competing interests

Financial disclosure: none for any of the authors.

## Authors’ contributions

KOG had full access to all of the data in the study and takes responsibility for the integrity of the data and the accuracy of the data analyses. Study concept and design*:* KOG, JDK, MA, CØ, HCS, and MS. Analysis and interpretation of the data: KOG, JDK, MA, CØ, HCS, and MS. Drafting of the manuscript: KOG. Critical revision of the manuscript for important intellectual concepts: KOG, JDK, MA, CØ, HCS, and MS. Statistical analysis: KOG. Administrative, technical, and material support: KOG, JDK, MA, CØ, HCS, and MS. Study supervision: KOG. All authors read and approved the final manuscript.

## Authors’ information

Contributing members of DACOBAN include Magnus Arpi, Kim O. Gradel, Ulrich S. Jensen (Copenhagen, Denmark), Jenny Dahl Knudsen, Kristoffer Koch (Aalborg, Denmark), Mette Pinholt (Herlev, Denmark), Henrik C. Schønheyder, Mette Søgaard, and Christian Østergaard.

## Pre-publication history

The pre-publication history for this paper can be accessed here:

http://www.biomedcentral.com/1471-2288/12/139/prepub
